# Comparison of Dexmedetomidine, Lidocaine, and Fentanyl in Attenuation Hemodynamic Response of Laryngoscopy and Intubation in Patients Undergoing Cardiac Surgery

**DOI:** 10.1155/2020/4814037

**Published:** 2020-07-01

**Authors:** Maziar Mahjoubifard, Mehdi Heidari, Maryam Dahmardeh, Seyed Bashir Mirtajani, Alireza Jahangirifard

**Affiliations:** ^1^Fellowship of Cardiac Anesthesia, Zahedan University of Medical Sciences, Zahedan, Iran; ^2^Mycobacteriology Research Center (MRC), National Research Institute of Tuberculosis and Lung Disease (NRITLD), Shahid Beheshti University of Medical Sciences, Tehran, Iran; ^3^Chronic Respiratory Disease Research Center, National Research Institute of Tuberculosis and Lung Diseases (NRITLD), Shahid Beheshti University of Medical Sciences, Tehran, Iran

## Abstract

**Materials and Methods:**

This clinical trial was conducted on 90 patients, aged 30–70 years, who had heart surgery. The participants were categorized into three groups. Group D received 1 *µ*g/kg intravenous dexmedetomidine in 10 minutes, group L received 1.5 mg/kg lidocaine (1%) 90 seconds before intubation, and group F received 2 *µ*g/kg fentanyl. The vital signs (HR, SBP, DBP, and MAP) were measured before intubation and 1st, 3rd, 5th, and 10th minutes after intubation. Data were analyzed with SPSS 19 software (chi-square, one-way ANOVA, or Kruskal–Wallis).

**Results:**

The age (*P*=0.389) and gender distributions of patients were similar in all three groups. Dexmedetomidine significantly attenuated HR in the 3^rd^ (*P*=0.001), 5^th^ (*P*=0.001), and 10^th^ (*P*=0.003) minutes after intervention. It also reduced the systolic blood pressure in the 5^th^ (*P*=0.024) and 10^th^ (*P*=0.006) minutes. This reduction was significantly higher in the dexmedetomidine group than that in the two other groups. In addition, dexmedetomidine caused a greater reduction in MAP in the 1^st^ (*P*=0.048), 5^th^ (*P*=0.0001), and 10^th^ (*P*=0.0001) minutes. *Discussion*. All three medications were effective in controlling HR; however, dexmedetomidine caused bradycardia in the 3^rd^, 5^th^, and 10^th^ minutes. Lidocaine resulted in an increase in MAP in the 1^st^ minute after intubation; whereas, dexmedetomidine reduced MAP at the 5^th^ and 10^th^ minutes after intubation. Changes in blood pressure and mean arterial pressure in the fentanyl group was lower than the two other groups.

**Conclusion:**

As a result, dexmedetomidine was not suitable for hemodynamic control and led to hypotension and bradycardia; on the other hand, fentanyl was more effective than two other medications in patients undergoing cardiac surgery. This trial is registered with IRCT2017013132320N1.

## 1. Introduction

Laryngoscopy and intubation are accompanied by sympathetic responses. These transient responses appear as an increase in blood pressure and heart rate, which appear immediately after intubation. These responses are due to an increase in the concentration of catecholamines in the plasma. Medications, such as fentanyl, esmolol, lidocaine, and *α*2-agonists, including clonidine and dexmedetomidine, have been used to reduce sympathetic responses to laryngoscopy and intubation [1–3].

In patients with cardiovascular disease, the hemodynamic changes may lead to life-threatening risks, such as heart ischemia, acute heart failure, and cerebrovascular events [4]. The intensity of laryngoscopy and intubation reflexes depends on the anesthetic depth, age, and complications, such as diabetes and heart diseases, of the patients [5].

Lidocaine is an aminoethyl amide, a member of the amide-type local anesthetic group. A single intravenous dose of lidocaine (1.5 mg/kg) was administered three minutes before intubation to attenuate hemodynamic responses to laryngoscopy and intubation, which produced desirable results [6–9].

Dexmedetomidine, as an *α*2-agonist, is 8 times more specific for *α*2 receptors than clonidine [1]. It reduced the sympathetic activity of the central nervous system and showed analgesic effects [6]. In addition, dexmedetomidine is used as a sedative for monitored anesthesia care, due to having analgesic and sedative impacts and no respiratory depression [10–12].

Although fentanyl has no significant effect on the cardiovascular system, it depends on the central vagus stimulation [13, 14]. Opioids neutralize the hemodynamic responses to intubation and surgical stresses, but are associated with nausea, vomiting, and hypoventilation [15]. There are many studies into the reduction of hemodynamic responses to laryngoscopy and intubation, and many medications have been used, but there is still no specific medication for it [16].

Effective measures have been taken to find a suitable remedy with the least destructive effects on heart patients under laryngoscopy. Thus, the current study was conducted to investigate the effect of fentanyl, lidocaine, and dexmedetomidine on patients with cardiac diseases, for whom the stability of hemodynamic conditions is very important, given the lack of a comparative study into these medications and the ineffectiveness or low effectiveness of fentanyl and lidocaine reported by some studies, despite their common use to reduce the laryngoscopy- and intubation-induced sympathoadrenal responses.

## 2. Methodology

This study is a clinical trial and was registered with code IRCT2017013132320N1.

The conduction of the study was initiated after final approval of the project by the Research Council of the Medical School of Zahedan Medical Sciences University, in coordination with the Operation Department of the Ali ibn Abi Taleb Hospital, and obtaining the informed written consent of eligible patients.

This clinical trial was conducted on 90 patients, aged 30–70 years, with ASA II and III, undergoing heart surgery under general anesthesia. The number of patients was calculated based on the following formula:

(1) $$\begin{array}{c} Z_{1-\left(\alpha /2\right)}=1.96,\\ Z_{1-\beta }=0.85,\\ X_{1}\pm S_{1}=94.62\pm 4.45,\\ X_{2}\pm S_{2}=91.07\pm 5.27,\\ N={\left(Z_{1-\left(\alpha /2\right)}+Z_{1-\beta }\right)}^{2}\frac{\left(S_{1}^{2}+S_{2}^{2}\right)}{{\left(X_{1}\_X_{2}\right)}^{2}}=30. \end{array}$$

The confidence level was 95%, and the test power was 80%.

The patients with hypotension (Sys Bp <90 mm Hg), bradycardia (HR <60 min), and severe LV dysfunction (EF <35%) were excluded from the study. These participants were using the simple random sampling technique and placed in three groups using the randomized selected block design.

**Figure 1:**
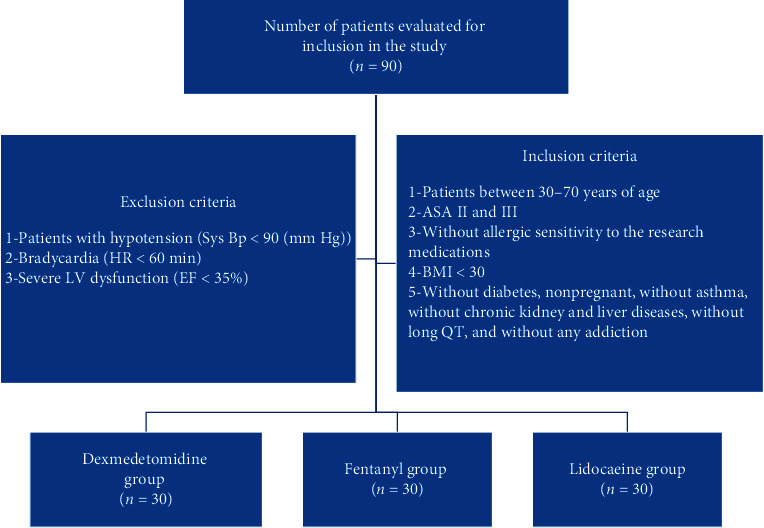
Consort chart.

Informed consent of patients was obtained, and all ethical codes were adhered. All patients were examined before anesthesia. Data such as the history of heart surgery, allergy comorbidities, and addiction were recorded. Clinical examinations of the heart and lungs were carried out and the Mallampati score was obtained. Inclusion criteria were patients between 30–70 years of age, with ASA II and III, without allergic sensitivity to the research medications, BMI <30, without diabetes, nonpregnant, without asthma, without chronic kidney and liver diseases, without long QT, and without any addiction. Exclusion criteria were intubation longer than 15 seconds and any intubation-related problem (Figure 1).

Patients were randomly assigned in three groups, each containing 30 participants. Group D received 1 *µ*g/kg intravenous dexmedetomidine (Behestan Daru Co., Iran) for 10 minutes before laryngoscopy [6]. Group L received 1.5 mg/kg intravenous lidocaine (1%; Tamin Co., Rasht, Iran), and group F received 2 *µ*g/kg fentanyl (Abureihan Co., Tehran, Iran) 90 seconds before intubation [5]. Since lidocaine is used routinely for the majority of patients, this group was regarded as the control. The operation team, nurses, patients, and anesthesia care team were blinded to this categorization. An outsider anesthetist prepared the medication-contained syringes and marked them with random codes.

After admission to the operation room, a 20-gauge angiocatheter was fixed for each patient and 3 mL/kg Ringer's serum or normal saline was infused. Then, the radial artery of the left hand was cannulated with a 20-gauge angiocatheter under sterilized conditions. The monitoring (SADAT, Alborz B5), including pulse oximetry (PO) and electrocardiogram (ECG), and capnography were carried out, and the baseline HR, SBP, DBP, and MAP were recorded. Patients received preoxygenation for 3 minutes at an O_2_ flow rate of 5 L/min.

Anesthesia was induced with etomidate in a dose of 0.3 mg/kg. In addition, a skeletal muscle relaxant (cisatracurium) in a dose of 0.15 mg/kg was administered to facilitate intubation.

Laryngoscopy was carried out by an experienced anesthesiologist using a MAC 3- or 4-bladed laryngoscope, based on the patient anatomy. Then, the trachea was intubated with a cuffed endotracheal tube with an appropriate internal diameter. The vital signs (HR, SBP, DBP, and MAP) were measured and recorded at the 1^st^, 3^rd^, 5^th^, and 10^th^ minutes after the intubation. A 20% increase in HR and/or MAP above the baseline was regarded as the positive response to intubation.

In compliance with the ethical guidelines, all research items were recorded on a form and explained to the participants. After ensuring a complete understanding of ethical items, consent form by the participants was taken.

Research limitations included death and medicinal complications. The sampling process continued until the desirable sample size was achieved.

## 3. Statistical Analyses

Descriptive statistics, mean, standard deviation, and percent, were used to data presentation. Variables normality distribution determined using the Kolmogorov–Smirnov test. One way ANOVA (if data had normal distribution) and Tukey's post hoc test or Kruskal–Wallis test (if data had not normal distribution) were used to compare quantity variables and the chi-square test for quantitate variables. All analyses were performed by SPSS software package version 19 (SPSS Inc., Chicago, IL). A value of *P* < 0.05 was considered to be significant.

## 4. Results

This study was conducted on 90 eligible inpatients, aged 30–70 years, undergoing heart surgery in Imam Ali Hospital. They were categorized into three groups, each containing 30 participants, after obtaining their informed written consent. According to the data in Table 1, 41 patients had an underlying disease (7 cases had diabetes and 34 had HTN). The age (*P*=0.389) and gender distributions were similar in all three groups (*P* > 0.05). Thirty patients in the dexmedetomidine group had Coronary Artery Bypass Grafting (CABG). In the fentanyl group, 27 patients had CABG and 3 patients underwent mitral valve replacement (MVR). In the lidocaine group, 28 patients had CABG, one patient underwent MVR, and one patient got AS + MS.

**Figure 2:**
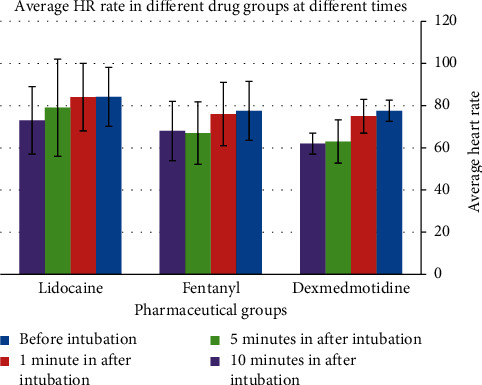
Comparison of HR in the 1st, 3rd, 5th, and 10th minutes after intubation with before intubation in each group.

The intergroup comparison of HR showed that the effect of dexmedetomidine on heart rate attenuation in the 3^rd^, 5^th^, and 10^th^ minutes was significantly stronger than in the two other medicines (Table 2). On the other hand, increased heart rate was not observed in the fentanyl and lidocaine groups (Figure 2).

**Figure 3:**
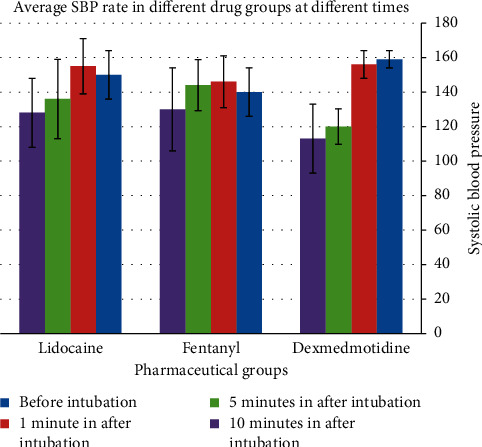
Comparison of SBP in the 1st, 3rd, 5th, and 10th minutes after intubation with before intubation in each group.

Dexmedetomidine reduced systolic blood pressure in the 1^st^, 3^rd^, 5^th^, and 10^th^ minutes (Table 3). This reduction was significantly higher as compared to that of the two other groups in the 5^th^ (*P*=0.001) and 10^th^ (*P*=0.003) minutes. A slight increase was observed in the mean systolic blood pressure of the patient in the lidocaine and fentanyl groups (Figure 3).

**Figure 4:**
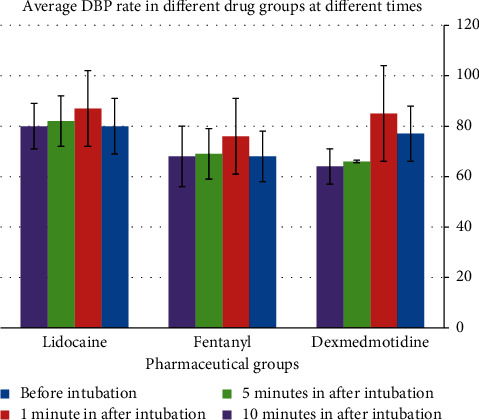
Comparison of DBP in the 1st, 3rd, 5th, and 10th minutes after intubation with before intubation in each group.

The intergroup comparison of the mean diastolic blood pressure showed the highest reduction in the dexmedetomidine group in the 5^th^ and 10^th^ minutes (Table 4). The mean diastolic blood pressure in the dexmedetomidine group reduced from the 3^rd^ minute onward (Figure 4).

The intergroup comparison of the mean arterial pressure showed that the dexmedetomidine was significantly more effective than the two other medicines in reducing MAP in the 1^st^, 5^th^, and 10^th^ minutes after intubation (Table 5). Dexmedetomidine attenuated MAP in the 3^rd^ minute, but not more significantly than the two other groups.

**Figure 5:**
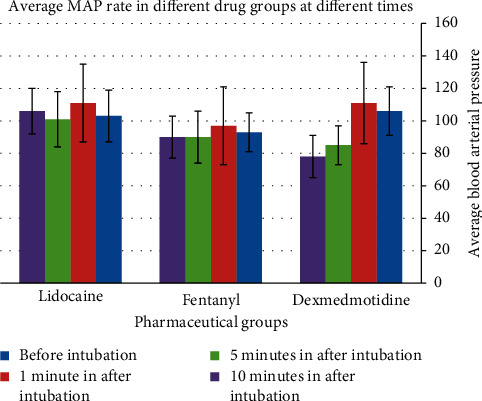
Comparison of MAP in the 1st, 3rd, 5th, and 10th minutes after intubation with before intubation in each group.

The comparison of mean arterial pressure before and after intubation in each group (Figure 5) showed a slight increase in MAP in all three groups in the 1^st^ minute; however, this increase was significant only in the lidocaine group (*P* < 0.02). According to these findings, fentanyl and dexmedetomidine were more effective than lidocaine in attenuating the responses to laryngoscopy and intubation in the 1^st^ minute.

Comparison of the mean arterial pressure in the 3^rd^ minute after intubation with the baseline showed a reduction only in the dexmedetomidine group; however, this reduction was not significant. With respect to the two other groups, a slight insignificant increase was observed in MAP (Figure 5).

The MAP in the 5^th^ minute after intubation was compared with baseline. Although MAP reduced in all three groups, this reduction was only significant in the dexmedetomidine group (*P* < 0.001). Dexmedetomidine in the 5^th^ minute attenuated the hemodynamic responses to laryngoscopy and intubation, and this reduction was greater in this group than the two other two groups (Figure 5). The MAP in the 10^th^ minute after intubation was compared with the baseline. Although MAP attenuation was observed in all three groups, it was only significant in the dexmedetomidine group (*P* < 0.001). Dexmedetomidine in the 10^th^ minute reduced the hemodynamic responses to laryngoscopy and intubation, and this effect was greater than the two other groups (Figure 5).

In Table 6, the numerical values of the effect of drugs are given relative to the base value of each of them. Based on the results of this study, changes in the dexmedetomidine group, in hemodynamic parameters (HR, SBP, and MAP), were more than 20% higher than the baseline values.

## 5. Discussion

The results of this study, in order to evaluate the effect of dexmedetomidine on patients undergoing laryngoscopy compared with other two drugs (fentanyl and lidocaine), showed no increase in HR as compared to the baseline in none of the three groups. In addition, dexmedetomidine significantly reduced HR in the 3rd, 5th, and 10th minutes after intubation as compared to the baseline. As compared to the two other medicines, dexmedetomidine led to a greater reduction in the systolic blood pressure in the 1st, 3rd, 5th, and 10th minutes and in the diastolic blood pressure in the 3rd, 5th, and 10th minutes after intubation. There was a significant difference between the dexmedetomidine group and the two other groups in the systolic and diastolic blood pressure in the 5th and 10th minutes after intubation. The MAP reduction was also higher in the dexmedetomidine group than the two other groups in the 3rd, 5th, and 10th minutes. This reduction was significant in the 5th and 10th minutes after intubation than the baseline. The MAP increased in the 1st and 3rd minutes relative to the baseline; however, this increase was significant only in the lidocaine group in the 1st minute.

DAS et al. [3], Ghorbanlo et al. [13], Gogus et al. in 2013 [11], Rani et al. in 2016 [17], and Gunalan et al. in 2015 [18] compared the effectiveness of fentanyl and dexmedetomidine in attenuating responses to laryngoscopy and intubation. According to the DAS results, dexmedetomidine was more effective than the fentanyl in preventing HR increase; however, fentanyl caused lower hypotension. According to Kataria et al. and Gunalan et al., dexmedetomidine was more effective than fentanyl in controlling the HR and MAP during laryngoscopy and intubation. In the current study, both dexmedetomidine and fentanyl reduced laryngoscopy- and intubation-induced responses; however, bradycardia and hypotension were higher in the dexmedetomidine group. Gogus et al. compared the effectiveness of dexmedetomidine (1 *µ*g/kg), fentanyl (2 *µ*g/kg), and esmolol (2 mg/kg) in preventing hemodynamic response to intubation. In the 5th and 10th minutes after intubation, HR in the dexmedetomidine group and DBP, SBP, and MAP in the esmolol group were lower than those in other groups [16]. This HR reduction in the dexmedetomidine was consistent with that in the current study. In Rani et al.‘s study, blood pressure reduced in both groups five minutes after intubation, but HR increased in the fentanyl group [17]. Inconsistent with Rani's study, blood pressure reduction and HR increase were not observed in the fentanyl group in the current study.

Reddy et al. compared dexmedetomidine (1 *µ*g/kg) and esmolol (2 mg/kg) and concluded that both medications were effective in attenuating responses to laryngoscopy; however, dexmedetomidine was more effective than esmolol [4]. The current study showed that dexmedetomidine prevented SBP and HR increase. In this group, an insignificant increase was observed in DBP and MAP only in the 1st minutes.

Gulabani et al. compared three groups, each containing 30 participants: lidocaine (1.5 mg/kg), dexmedetomidine (0.5 *µ*g/kg), and dexmedetomidine (1 *µ*g/kg). They examined hemodynamic parameters before and 1, 3, and 5 minutes after intubation and concluded that dexmedetomidine in a dose of 1 *µ*g/kg was the most effective medicine in reducing these responses [6]. According to the findings, lidocaine was also effective in reducing the responses, except in the 1st minute when MAP increased. Compared to preintubation, this difference was significant.

Soltani Mohammadi et al. compared oral lidocaine (0.2 mg 90 minutes before intubation) and intravenous lidocaine (1.5 mg/kg) and did not observe any difference between them; however, the incidence of such complications as bradycardia and orthostatic was higher in the lidocaine group [5]. According to the findings of the current study, the incidence of bradycardia and hypotension was higher in the group treated with dexmedetomidine, as an *α*2-agonist.

Hassani et al. compared the effectiveness of fentanyl (2 *µ*g/kg) and fentanyl (2 *µ*g/kg) + lidocaine (1.5 mg/kg) in reducing hemodynamic responses to tracheal intubation. They showed that fentanyl + lidocaine was more effective than fentanyl alone [19]. In the current study, fentanyl was more effective than lidocaine. In addition, a significant increase in blood pressure was observed one minute after intubation. Gurulingappu et al. investigated the effectiveness of fentanyl (4 *µ*g/kg) and lignocaine (1.5 mg/kg) in weakening cardiovascular responses to direct laryngoscopy and intubation. Although pressure attenuation was observed in both groups, it was greater in the fentanyl group [15]. This finding was consistent with that of the current study. Min et al. compared the effectiveness of remifentanil (10 *µ*g/kg) with lidocaine (1.5 mg/kg) + esmolol (1 mg/kg) in the attenuation of hemodynamic response to laryngoscopy and intubation. Results showed that remifentanil was more effective, and MAP changes were greater in the lidocaine + esmolol group than in the remifentanil group.[20].

Sarkar et al. compared the effectiveness of intravenous clonidine (3 *µ*g/kg) and dexmedetomidine (0.5 *µ*g/kg) in reducing blood pressure caused by laryngoscopy and intubation. Results suggested the effectiveness of clonidine and dexmedetomidine in this regard. The postintubation SBP was lower in both groups, but HR was higher in the dexmedetomidine group [21]. In contrast, HR was lower in the dexmedetomidine group in the current study.

Rashmi and Komala in 2015 [22], Laha et al. in 2011 [23], and Kumari et al. in 2008–2009 [24] evaluated the effect of intravenous dexmedetomidine on hemodynamic responses to laryngoscopy and intubation. In these three studies, HR, DBR, SBP, and MAP reduced in the dexmedetomidine group, indicating its effectiveness in attenuating hemodynamic response to laryngoscopy and intubation. Also, based on the specificity of dexmedetomidine (hypotension, bradycardia, etc.), in many cases, hemodynamic changes in patients after injection of this drug are severe (>20%). Shu and his colleagues also acknowledged this in 2019 [25].

## 6. Conclusions

It is known that a change more than 20–25% in BP and to some extent in HR from the baseline may be harmful. So, looking for this change is important even though the values are in the normal ranges. In our study, we could find the mentioned changes in the dexmedetomidine group.

All three medications were effective in controlling HR; however, dexmedetomidine caused bradycardia in the 3rd, 5th, and 10th minutes. Lidocaine resulted in an increase in MAP 1 minute after intubation, whereas dexmedetomidine attenuated MAP at 5th and 10th minutes after intubation. Changes in HR and MAP were lower in the fentanyl group, and this medication was more effective in suppressing the hemodynamic responses to laryngoscopy and intubation in patients undergoing heart surgeries because changes in HR and MAP after induction were more in the lidocaine group than fentanyl one. Also, MAP increased more in the lidocaine group at the first time of registration, so we can say that fentanyl is more effective than lidocaine in keeping BP more stable.

## Figures and Tables

**Table 1:** Demographic information of participants.

	Gender		Age	Height	Underlying disease	
Groups	Female	Male	Mean ± SD	Mean ± SD	HTN	Diabetes
Dexmedetomidine	13	17	60.43 ± 7.62	165.67 ± 12.06	13	3
Fentanyl	16	14	57.27 ± 11.34	165.90 ± 9.65	8	2
Lidocaine	12	18	60.37 ± 11.05	165.17 ± 9.65	13	2
*P* value	0.421	0.506	0.389	0.963	NA	NA

**Table 2:** Intergroup comparison of HR (meaningful level *P* <  0.05.

	HR before intubation	HR in the 1^st^ min	HR in the 3^rd^ min	HR in the 5^th^ min	HR in the 10^th^ min
	Mean ± SD	Mean ± SD	Mean ± SD	Mean ± SD	Mean ± SD
Dexmedetomidine	77.63 ± 10.33	75.26 ± 8.32	66.67 ± 10.79	63.77 ± 5.97	62.03 ± 5.04
Fentanyl	78.53 ± 14.83	76.90 ± 15.30	71.4617.27	67.03 ± 14.74	68.57 ± 14.09
Lidocaine	84.20 ± 20.35	84.00 ± 16.33	83.53 ± 16.10	79.20 ± 14.03	73.73 ± 16.87
*P* value	0.22	0.038	0.001	0.001	0.003

**Table 3:** Comparison of systolic blood pressure in three groups in the 1st, 3rd, 5th, 10th minutes after intubation with before intubation (meaningful level *P* < 0.05).

	SBP in before intubation	SBP in the 1^st^ min	SBP in the 3^rd^ min	SBP in the 5^th^ min	SBP in the 10^th^ min
	Mean ± SD	Mean ± SD	Mean ± SD	Mean ± SD	Mean ± SD
Dexmedetomidine	159.07 ± 22.21	156.77 ± 34.64	138.90 ± 38.78	120.80 ± 22.34	113.50 ± 20.62
Fentanyl	140.30 ± 27.48	146.70 ± 37.49	144.00 ± 33.80	134.10 ± 23.69	130.57 ± 24.47
Lidocaine	150.07 ± 25.26	155.07 ± 38.02	137.53 ± 23.43	136.20 ± 23.49	128.80 ± 20.46
*P* value	0.018	0.527	0.722	0.024	0.006

**Table 4:** Comparison of diastolic blood pressure in three groups in the 1st, 3rd, 5th, and 10th minutes after intubation with before intubation (meaningful level *P* < 0.05).

	DBP in before intubation	DBP in the 1^st^ min	DBP in the 3^rd^ min	DBP in the 5^th^ min	DBP in the 10^th^ min
	Mean ± SD	Mean ± SD	Mean ± SD	Mean ± SD	Mean ± SD
Dexmedetomidine	77.53 ± 9.52	85.60 ± 19.90	76.33 ± 16.68	66.80 ± 11.13	64.26 ± 7.81
Fentanyl	68.60 ± 10.25	76.93 ± 15.43	76.23 ± 12.57	69.90 ± 10.90	68.33 ± 12.30
Lidocaine	80.53 ± 10.27	87.40 ± 15.44	80.27 ± 8.88	82.40 ± 11.05	80.80 ± 9.71
*P* value	0.001	0.044	0.40	0.001	0.001

**Table 5:** Intergroup comparison of the mean arterial blood pressure (meaningful level *P* < 0.05).

	MAP in before intubation	MAP in the 1^st^ min	MAP in the 3^rd^ min	MAP in the 5^th^ min	MAP in the 10^th^ min
	Mean ± SD	Mean ± SD	Mean ± SD	Mean ± SD	Mean ± SD
Dexmedetomidine	106.10 ± 12.99	111.03 ± 25.14	98.600 ± 28.98	85.70 ± 15.37	78.97 ± 13.46
Fentanyl	93.00 ± 16.48	97.66 ± 24.57	98.43 ± 19.54	90.93 ± 12.08	90.13 ± 13.70
Lidocaine	103.13 ± 17.65	111.87 ± 24.13	103.67 ± 15.60	101.47 ± 16.92	96.47 ± 14.08
*P* value	0.005	0.048	0.58	0.0001	0.0001

**Table 6:** The rate of change in the effectiveness of dexmedetomidine, fentanyl, and lidocaine compared to baseline values in each of the four hemodynamic indexes (HR, SBP, DBP, and MAP).

Hemodynamic factors	Groups	In before intubation (baseline)	In the 1^st^ min	In the 3^rd^ min	In the 5^th^ min	In the 10^th^ min
HR	Dexmedetomidine	0	−3.05	−14.11	−17.85	−20.09^*∗*^
	Fentanyl	0	−2.07	−9.00	−14.64	−12.68
	Lidocaine	0	−0.23	−0.79	−5.93	−12.43

SBP	Dexmedetomidine	0	−1.44	−12.67	−24.05^*∗*^	−28.64^*∗*^
	Fentanyl	0	+4.56	+2.63	−4.41	−6.93
	Lidocaine	0	+3.33	−8.35	−9.24	−14.17

DBP	Dexmedetomidine	0	+10.40	−1.54	−13.83	−17.11
	Fentanyl	0	+12.14	+11.12	+1.89	−0.39
	Lidocaine	0	+8.53	−0.32	+2.32	+0.33

MAP	Dexmedetomidine	0	+0.98	−7.06	−19.22	−25.57^*∗*^
	Fentanyl	0	+5.01	+5.83	−2.22	−3.08
	Lidocaine	0	+8.47	+0.52	−1.60	−6.45

(+) Means the relative increase of numbers, and (−) means the relative decrease of the variations relative to the baseline value. In cases with^*∗*^, changes are more than 20%.

## Data Availability

No data were used to support this study.
